# Moving Toward “Laboratory-Supported” Criteria for Psychogenic Tremor

**DOI:** 10.1002/mds.23922

**Published:** 2011-09-28

**Authors:** Petra Schwingenschuh, Petra Katschnig, Stephan Seiler, Tabish A Saifee, Maria Aguirregomozcorta, Carla Cordivari, Reinhold Schmidt, John C Rothwell, Kailash P Bhatia, Mark J Edwards

**Affiliations:** 1 Sobell Department of Motor Neuroscience and Movement Disorders, UCL Institute of NeurologyLondon, United Kingdom; 2 Department of Neurology, Medical University of GrazGraz, Austria; 3 Neurophysiology Department, National Hospital for Neurology and NeurosurgeryLondon, United Kingdom

**Keywords:** psychogenic tremor, tremor analysis, electrophysiology, diagnostic criteria

## Abstract

A confident clinical diagnosis of psychogenic tremor is often possible, but, in some cases, a “laboratory-supported” level of certainty would aid in early positive diagnosis. Various electrophysiological tests have been suggested to identify patients with psychogenic tremor, but their diagnostic reliability has never been assessed “head to head” nor compared to forms of organic tremor other than essential tremor or PD. We compared baseline tremor characteristics (e.g., frequency and amplitude) as well as electrophysiological tests previously reported to distinguish psychogenic and organic tremor in a cohort of 13 patients with psychogenic tremor and 25 patients with organic tremor, the latter including PD, essential-, dystonic-, and neuropathic tremors. We assessed between-group differences and calculated sensitivity and specificity for each test. A number of tests, including entrainment or frequency changes with tapping, pause of tremor during contralateral ballistic movements, increase in tremor amplitude with loading, presence of coherence, and tonic coactivation at tremor onset, revealed significant differences on a group level, but there was no single test with adequate sensitivity and specificity for separating the groups (33%–77% and 84%–100%, respectively). However, a combination of electrophysiological tests was able to distinguish psychogenic and organic tremor with excellent sensitivity and specificity. A laboratory-supported level of diagnostic certainty in psychogenic tremor is likely to require a battery of electrophysiological tests to provide sufficient specificity and sensitivity. Our data suggest such a battery that, if supported in a prospective study, may form the basis of laboratory-supported criteria for the diagnosis of psychogenic tremor. © 2011 Movement Disorder Society

Psychogenic tremor (PsyT) is the most commonly reported psychogenic movement disorder (PMD).^1^ Current diagnostic criteria for PMD^2^ are based, at least in part, on clinical findings that are incongruent with organic movement disorders; with regard to tremor, these include distractibility and entrainment. However, there is no gold standard for diagnosing PsyT apart from these clinical criteria. In some patients, uncertainty remains and may result in diagnostic delay and unnecessary investigation and treatment. The importance of a positive diagnosis, rather than one of exclusion, has been repeatedly emphasized,^3^ and in line with this, it has been suggested that a “laboratory-supported” level of certainty might be added to current clinical diagnostic criteria.^4^

A variety of electrophysiological tests have been proposed as useful in distinguishing PsyT from organic tremor (OrgT),^5^, ^6^ and might therefore qualify for inclusion in this new level of diagnostic certainty. Previously reported characteristics of PsyT include a tonic discharge of antagonist muscles approximately 300ms before the onset of tremor bursts,^3^ an increase of tremor amplitudes in response to weighting the limb,^3^, ^7^ entrainment or an increase in variability and change of tremor frequency while tapping with the contralateral hand,^7^–^10^ less accurate tapping performance at requested frequencies,^7^ significant coherence in bilateral tremors,^11^ and transient arrest of tremor during a ballistic movement of the other hand.^12^

Current data, using such tests to distinguish between PsyT and OrgT, have two important limitations. First, there has been no direct head-to-head comparison of the utility of these techniques in the same sample of patients. Second, and perhaps more important, most tests have only been compared between PsyT and essential tremor (ET) or PD tremor, rather than more unusual OrgT disorders, such as dystonic tremor (DT), where differentiation from PsyT may be more difficult.

In this study, we, therefore, set out to directly compare the sensitivity and specificity of these electrophysiological techniques in a group of patients with different tremor disorders. We consider this study a first step toward the development of laboratory-supported criteria for PsyT.

## Patients and Methods

### Patients

Thirteen patients with PsyT (mean age: 47.1 ± 15.0 years; mean disease duration: 8.2 ± 6.3 years) and 25 patients with OrgT (mean age: 67.5 ± 14.3 years; mean disease duration: 13.8 ± 15.1 years) were studied. The OrgT group consisted of 9 patients with PD, 9 with DT, 5 with ET, and 2 with neuropathic tremor (NT). PsyT and OrgT were classified according to published criteria.^2^, ^13^ All patients were diagnosed by two movement disorder experts (M.J.E. and K.P.B.). We selected a clinically heterogeneous PsyT group that included 8 patients who did not show certain clinical criteria—such as entrainability and distractibility—that would be relevant for the outcome of some of the electrophysiological tests, but in whom diagnosis was based on other criteria, such as presence of further somatizations and placebo response. Demographics and clinical characteristics of PsyT patients are given in Table 1. Local Research Ethics Committee approval was obtained, and all patients gave informed consent.

**TABLE 1:** Demographics and Clinical Characteristics of the 13 Patients With Psychogenic Tremor

	Sex	AAO	DD	Acute Onset	Spontaneous Remissions	Placebo Response	Remission with Psychotherapy	Absent When Unobserved	Underlying Psychopathology	FH	Other Somatizations	Tremor Distribution	Distract ability*	Entrain ment	Unusual Variability	Coactiva tion	Other Features	Level**
P1	F	53	1	Y	N	Y	N	N	MLE	N	N	RA, LA	Y	N	Y	Y	Uses wheelchair, fatigue	A
P2	M	68	3	Y	N	Y	N	N	N	N	N	RA, LA	Y	N	N	N	N	A
P3	F	22	8	Y	N	Y	N	N	N	N	DA, UP	RA, LA, LL	N	N	Y	N	Unusual parkinson-like gait	A
P4	F	17	2	N	N	NA	N	N	ED	N	N	H, RA, LA	N	N	N	Y	N	B
P5	F	48	6	Y	N	Y	N	N	D	N	N	RA, LA, RL, LL	N	N	Y	Y	Unusual gait	A
P6	M	60	4	Y	N	Y	N	N	D	N	N	RA, RL	Y	Y	N	Y	Unusual gait	A
P7	M	39	7	N	N	Y	N	N	N	N	N	H, RA	Y	N	N	N	Unusual gait and slowness	A
P8	F	33	3	N	N	Y	N	N	ED, S	N	N	RA, LA	N	N	Y	N	N	A
P9	F	18	11	Y	N	Y	N	N	D	N	UP, UV	RA, LA, RL	N	N	Y	Y	N	A
P10	M	34	20	N	Y	NA	N	N	N	N	N	RA, LA	N	N	Y	Y	Unsteadiness	B
P11	F	35	17	Y	N	Y	N	N	D	N	UP, UB	RA, LA, RL, LL	N	N	Y	Y	Unusual gait, uses wheelchair	A
P12	M	41	4	Y	N	NA	N	N	N	N	N	LA	N	N	Y	Y	Give-way weakness	B
P13	M	39	18	Y	N	Y	N	N	N	N	N	LA	Y	N	Y	Y	Position-specific tremor	A

P1-13, psychogenic tremor patients.

* Distractibility (with counting backward, arithmetics, performing a tapping sequence with the other hand, and recognizing a letter being written by the examiner on the patient's back). AAO, age at onset in years; DD, disease duration in years; Y, yes; N, no; NA, not applicable; placebo response, a nonphysiological (placebo) response to botulinum toxin injections, defined as tremor cessation immediately after injections lasting more than 1 hour; MLE, major life event before tremor onset, such as sexual abuse; D, depression; ED, eating disorder; S, history of self-harm; AD, anxiety disorder; FH, positive family history for tremor or another movement disorder in a first-degree relative; DA, *Dermatitis artefacta*; UP, medically unexplained pain disorder; UV, medically unexplained vomiting; UB, medically unexplained bladder dysfunction requiring self-catheterization; H, head; RA, right arm; LA, left arm; RL, right leg; LL, left leg; T, trunk.

** Level of diagnostic certainty of psychogenic tremor according to current clinical diagnostic criteria (A: documented; B: clinically established; C: probable; D: possible).^2^

### Accelerometry and EMG

Patients were comfortably seated in a chair. A triaxial accelerometer transducer (sensitivity ± 100 mV/G, Biometrics ACL300; Biometrics Ltd., Cwmfelinfach, Wales, UK) was attached to the dorsal surface of the middle phalanx of the index finger bilaterally. Surface electromyography (EMG) was recorded from wrist flexors (WFs) and wrist extensors (WEs). EMG signals were amplified (Digitimer Ltd., Welwyn Garden City, UK) and analog filtered (low pass at 1,000 Hz and high pass with 3-ms time constant) and sampled at 2,000 Hz.

Recordings were performed (1) with arms relaxed and hands hanging freely from the arm rest (rest), (2) with arms/wrists outstretched at shoulder level without (posture), and (3) with a 500-g mass attached to the wrists, (4) while performing a goal-directed task (action), (5) during a tapping task, and (6) while performing ballistic hand movements. For the tapping task, subjects were instructed to use the less-affected hand to tap in time with a metronome at rates of 1, 3, and 5 Hz while ignoring the more symptomatic arm. For the ballistic movement task, subjects were instructed to point with the index finger of an outstretched arm at shoulder level at the examiner's index finger and follow it as fast as possible when the examiner would change position abruptly. During the tasks, EMG and accelerometry were recorded continuously from the more affected contralateral arm in the position (i.e., rest or posture) where tremor was maximal. The tremor was recorded and analyzed for 30 seconds in each condition.

### Data Analysis and Statistics

A Fourier analysis of the signals derived from accelerometry was performed to define peak tremor frequency (PF), total power of the spectra between 1 and 30 Hz as a measure of tremor amplitude (TP), half-width power (HWP) given by the area under the curve between two vertical straight lines intersecting the rising, and falling edge of the peak at half peak power (full-width half maximum; FWHM), with the latter being a measure of frequency stability/variability.^14^ All parameters were calculated for each accelerometer axis, then averaged.

EMG recordings were used offline to define the onset of each ballistic movement. The effect of ballistic hand movements was assessed, as previously described,^12^ by assessing changes in tremor amplitude and frequency as well as latency and duration of significant changes. Tremor was considered sensitive to the execution of a contralateral ballistic movement in patients in whom a tremor pause or at least a 50% decrease in period or amplitude occurred in 7 or more of 10 trials.^12^

We performed coherence and phase analysis on rectified EMG signals from WE and WF of the tremor dominant arm to define whether EMG bursts occurred simultaneously (i.e., in phase), in an alternating pattern (i.e., 180 degrees out of phase), or somewhere between. In the 32 of 38 subjects with a bilateral hand tremor, we additionally assessed coherence between EMG of right and left WE. The presence of coherence was defined as two contiguous bins on the coherence plot that rose above the 99% confidence limit for random coherence at a frequency where there were corresponding peaks in the power spectra.^9^ Analyses of tapping task performance and entrainment are explained more fully below. We measured and averaged the duration of tremor bursts for 30 seconds on WE-EMG recordings of postural tremor of the more affected arm. After anonymizing the data, we inspected the EMG of WE and WF of the tremor-dominant arm for the presence of a tonic coactivation phase at tremor onset (i.e., start of rhythmic oscillations on accelerometry). The tonic coactivation phase was defined as tonic discharge of antagonist muscles approximately 300 ms before the onset of tremor bursts.^3^

Statistical analysis was performed using PASW Statistics 18 (SPSS, Inc., Chicago, IL). Baseline tremor characteristics were compared between OrgT and PsyT by independent-samples *t*-test. To assess the effect of loading and tapping on PF and TP of the tremor-dominant hand, we performed a repeated-measures analysis of variance (ANOVA). All post-hoc comparisons were corrected by the Bonferroni method. For categorical data, group analysis was performed with 2 × 2 cross-tabs. We compared the frequency of abnormal test results by chi-square tests and Fisher's exact test (two-sided) and obtained sensitivity and specificity for each test. *P* values below 0.05 were considered to indicate statistical significance.

## Results

Baseline tremor characteristics are given in Table 2.

**TABLE 2:** Comparison of Baseline Tremor Characteristics in OrgT and PsyT

Significance level	PsyT	OrgT	*P* Value
Rest PF (Hz)	5.6 ± 1.5	5.1 ± 0.9	0.3
Rest TP (G^2^ )	0.00067 ± 0.00115	0.00032 ± 0.00059	0.4
Rest HWP (G^2^ )	0.00023 ± 0.00052	0.00009 ± 0.00020	0.3
Rest FWHM (G^2^ )	1.8 ± 0.3	2.2 ± 0.9	0.2
Posture PF (Hz)	5.9 ± 2.0	5.8 ± 1.0	0.9
Posture TP (G^2^ )	0.00151 ± 0.00235	0.00047 ± 0.00126	0.2
Posture HWP (G^2^ )	0.00036 ± 0.00044	0.00009 ± 0.00020	0.056
Posture FWHM (G^2^ )	2.0 ± 0.5	2.1 ± 0.9	0.6
Action PF (Hz)	6.3 ± 1.7	6.4 ± 1.5	0.8
Action TP (G^2^ )	0.00149 ± 0.00124	0.00041 ± 0.00041	<0.001*
Action HWP (G^2^ )	0.00059 ± 0.00049	0.00017 ± 0.00019	0.001*
Action FWHM (G^2^ )	3.2 ± 0.9	3.4 ± 1.2	0.5
EMG burst duration (ms)	93.1 ± 22.8	89.3 ± 21.2	0.6
Phase (degrees)	104.3 ± 72.1	99.4 ± 66.7	0.8

All values are given as mean ± standard deviation. A star indicates a significant difference between the groups at a 5% level.

Abbreviations: PsyT, psychogenic tremor group; OrgT, organic tremor group; PF, peak tremor frequency; TP, total power; HWP, half-width power; FWHM, full-width half maximum; Hz, hertz; G, gravity.

### 500-g Loading

We compared TP (accelerometry; more affected hand) of a 30-second epoch before and after loading. A repeated-measures ANOVA showed a significant interaction of loading and group (F_(1,36)_ = 4.69, *P* = 0.037) and a significant effect of group (F_(1,36)_ = 5.17, *P* = 0.029), which was explained by a significant increase of TP with loading in PsyT, compared to OrgT. We calculated the within-patient percent change in TP after loading and defined a cut-off value of abnormal increase of TP (mean of OrgT data + 2 SD [standard deviation]). The upper limit of normal, as defined above, was 130%, and 4 of 12 PsyT versus 2 of 25 OrgT (1DT, 1NT) had an increase of TP after loading above this level (*P* = 0.073; test sensitivity: 33%; test specificity: 92%).

### Tapping Task Performance

There was no significant difference in task performance on a group level. Average tapping frequencies (derived from 30-second epochs) were as follows: for 1-Hz tapping, 1.31 ± 1.48 Hz in PsyT and 1.01 ± 0.01 Hz in OrgT (*P* = 0.5); for 3-Hz tapping, 3.07 ± 0.39 Hz in PsyT and 3.09 ± 0.43 Hz in OrgT (*P* = 0.9); and for 5-Hz tapping, 4.37 ± 1.33 Hz in PsyT and 4.99 ± 0.38 Hz in OrgT (*P* = 0.1).

We defined correct tapping performance for 1, 3, and 5 Hz as 0.5 to 1.5 Hz, 2.5 to 3.5 Hz, and 4.5 to 5.5 Hz, respectively. The rate of incorrect performances was higher in the PsyT, compared to the OrgT, group, although this difference was not significant: 2 of 13 PsyT patients and 0 of 25 OrgT patients performed the 1-Hz tapping task incorrectly (*P* = 0.1); 3 of 13 PsyT patients and 1 of 25 OrgT patients (1 PD) performed the 3-Hz task incorrectly (*P* = 0.1); and 4 of 13 PsyT patients and 3 of 25 OrgT patients (1 PD, 2 NT) performed the 5-Hz task incorrectly (*P* = 0.2).

Incorrect task performance in at least one of the tapping frequencies was found in 6 of 13 PsyT and 4 of 25 OrgT patients (*P* = 0.062; test sensitivity: 46%; test specificity: 84%).

### Entrainment

At 1-Hz tapping, 1 of 13 PsyT patients had tremor suppression and another patient showed entrainment. At 3-Hz tapping, 1 of 13 PsyT patients had tremor suppression and 2 showed entrainment. At 5-Hz tapping, 3 of 13 PsyT patients showed entrainment. None of the 25 OrgT patients entrained to 1-, 3-, or 5-Hz tapping. Therefore, entrainment during contralateral tapping in at least one of three frequencies was found in 5 of 13 PsyT and 0 of 25 OrgT patients (*P* = 0.003; test sensitivity: 39%; test specificity: 100%).

### Frequency Shift During Tapping

To further evaluate the effect of finger tapping at requested frequencies of 1, 3, and 5 Hz on PF (derived from 30-second epochs each) of the contralateral hand, we performed a repeated-measures ANOVA with Tapping (baseline, 1 Hz, 3 Hz, and 5 Hz) as a within-subject factor and group (OrgT, PsyT) as between-subjects factors. There was a significant effect of tapping on the tremor frequency of the contralateral hand (F_(3,105)_ = 3.14; *P* = 0.029) and a significant tapping × group interaction (F_(3,105)_ = 3.79; *P* = 0.013). Post-hoc tests with a Bonferroni correction revealed this to be the result of a significant absolute reduction of tremor frequency while tapping at 3 Hz (*P* = 0.049) in the PsyT group (5.64 ± 2.04 Hz at baseline; 4.62 ± 2.24 Hz during 3-Hz tapping).

We calculated relative change of tremor frequency during 1-, 3-, and 5-Hz tapping, compared to baseline (Table 3), and defined the cut-off level for a pathological change of tremor frequency during contralateral tapping as mean + 2SD from OrgT data. Therefore, the upper limit of normal change in tremor frequency at 1 Hz was 19.0%, at 3 Hz was 26.9%, and at 5 Hz was 25.7%. Using the above cut-off values, 8 of 13 PsyT and 1 of 25 OrgT (1 PD) patients showed abnormal tremor frequency shift during 1-Hz tapping. Furthermore, 6 of 13 PsyT and 1 of 25 OrgT patients (1 DT) showed abnormal tremor frequency shift during 3-Hz tapping, whereas 5 of 13 PsyT and 1 of 25 OrgT patients (1 PD) showed abnormal tremor frequency shift during 5-Hz tapping. Therefore, an abnormal frequency shift during contralateral tapping in at least one of three frequencies was found in 10 of 13 PsyT and 3 of 25 OrgT patients (*P* < 0.001; test sensitivity: 77%; test specificity: 88%).

**TABLE 3:** Percent Change of Tremor Frequency During Tapping in PsyT Versus OrgT

	1-Hz Tapping (% Change Tremor Frequency)	3-Hz Tapping (% Change Tremor Frequency)	5-Hz Tapping (% Change Tremor Frequency)
PsyT (mean ± SD)	40.1 ± 38.6	29.6 ± 29.9	27.0 ± 27.7
OrgT (mean ± SD)	6.8 ± 5.6	8.7 ± 8.8	9.8 ± 7.9
*P* value	0.009	0.029	0.048

Abbreviations: PsyT, psychogenic tremor group; OrgT, organic tremor group; SD, standard deviation.

Only 3 PsyT patients had no abnormal shift in any of the tapping tasks. However, 2 of these patients had poor task performance in at least one of the requested frequencies. Hence, only 1 PsyT patient had a correct task performance on all three requested tapping frequencies without showing significant contralateral tremor frequency change.

### Ballistic Movement

One PsyT patient was excluded from analysis because she was not able to perform the task as instructed. Compared to baseline, at least a 50% decrease in period or amplitude after the contralateral ballistic movement in at least 7 of 10 trials^12^ occurred significantly more frequently in PsyT (8 of 12) than in OrgT (4 of 25: 2 PD, 1 DT, 1 ET) (*P* = 0.006; test sensitivity: 67%; test specificity: 84%). A clear pause in tremor was observed in at least 7 of 10 trials in 5 of 12 PsyT patients and in none of the OrgT patients (*P* = 0.002; test sensitivity: 42%; test specificity: 100%).

**FIG. 1:**
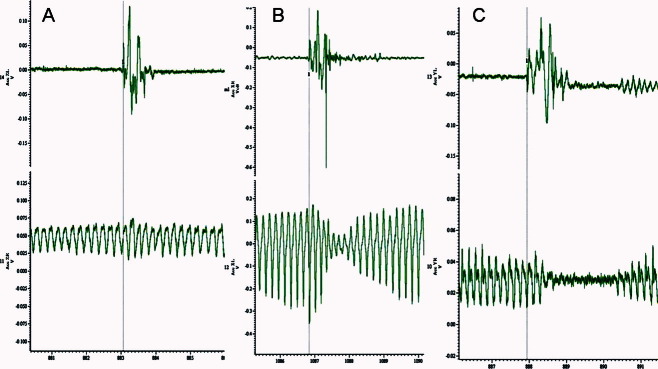
Representative examples of the effects of the ballistic movement test. Three representative examples of the effects of ballistic movement (upper traces on each pair; lower traces represent contralateral hand tremor): (A) Patient with OrgT (PD) and no effect on tremor; (B) patient with OrgT (PD) and >50% reduction of tremor amplitude; and (C) patient with PsyT and a pause of tremor.

In subjects with a significant decrement of tremor-related oscillations after contralateral ballistic movements, latency and duration were, on average, 1.02 ± 0.46 seconds and 1.88 ± 0.98 seconds in PsyT and 0.96 ± 0.75 seconds (*P* = 0.85) and 1.29 ± 0.76 seconds (*P* = 0.15) in OrgT. Representative examples of the effects of ballistic movement are given in Fig. 1.

### Coherence

Significant EMG coherence in bilateral arm tremors was found more frequently in PsyT (5 of 9) than in OrgT (1 of 23: 1 PD) (*P* = 0.003; test sensitivity: 56%; test specificity: 96%).

### Tonic Coactivation

One episode of tremor onset could be recorded in 11 of 13 PsyT and 23 of 25 OrgT patients. A tonic coactivation phase was found in 5 of 11 PsyT and in 1 of 23 OrgT (1 DT) patients (*P* = 0.008; test sensitivity: 46%; test specificity: 96%).

### Summary Score

Test sensitivity and specificity of all separate tests varied between 33% and 77% and 84% and 100%, respectively. To strengthen the discriminative value of the electrophysiological tests, we calculated a sum score for all performed tests (maximum 10 points).

- Incorrect tapping performance at 1, 3, and 5 Hz (maximum 3 points)
- Entrainment, suppression, or pathological frequency shift at 1, 3, and 5 Hz (maximum 3 points)
- Pause or >50% reduction in amplitude of tremor with ballistic movements (1 point)
- Tonic coactivation before tremor onset (1 point)
- Coherence of bilateral tremors (1 point)
- Increase of TP (as surrogate of tremor amplitude) with loading (1 point)

Patients with PsyT had a higher average score on the test battery, compared to patients with OrgT (3.9 ± 0.9 points versus 0.6 ± 0.8 points; *P* < 0.001). All OrgT patients had either 0 or 1 or 2 points (see Supporting Table 5). In contrast, all PsyT patients had 3 points or more (Table 4). We, therefore, defined the cut-off score for a diagnosis of laboratory-supported PsyT with 3 of 10 points (test sensitivity and specificity of 100%; *P* < 0.001).

**TABLE 4:** Results of the Test Battery in 13 Patients With Psychogenic Tremor

	Incorrect Tapping	Response to Tapping	Response to Ballistic Movements	Tonic Coactivation	Coherence	Response to Loading	Total Score (Maximum 10)
P1	2	3	NA	0	0	0	5
P2	0	3	1	0	0	0	4
P3	1	1	1	0	1	0	4
P4	0	2	0	1	1	0	4
P5	0	3	1	0	1	1	6
P6	0	3	1	NA	NA	NA	4
P7	1	1	1	0	NA	0	3
P8	2	0	1	0	0	0	3
P9	2	2	0	NA	0	0	4
P10	1	0	0	1	1	1	4
P11	0	1	0	1	1	1	4
P12	0	0	1	1	NA	1	3
P13	0	1	1	1	NA	0	3

P1-13, psychogenic tremor patients 1-13. NA (not applicable), in some items, indicates an inability to perform the task as instructed (response to ballistic movements), lack of a recording of tremor onset in continuous tremors (tonic coactivation), presence of a unilateral tremor only (coherence), or absence of a postural tremor (response to loading).

## Discussion

We have, for the first time, compared the discriminative value of previously developed electrophysiological tests to differentiate between PsyT and OrgT. Our data suggest that a number of tests are potentially useful to discriminate PsyT from a broad range of OrgT (e.g., PD, ET, DT, and NT), but on their own, they lack sensitivity, specificity, or both. However, a combination of tests was able to classify PsyT and OrgT more accurately and may, therefore, provide the basis for laboratory-supported criteria.

We confirm that simple tremor analysis (i.e., frequency and amplitude) is generally unhelpful for the differentiation of PsyT from OrgT.^3^ However, as obsreved in two previous studies, which included patients with PD and ET,^3^, ^7^ change in tremor amplitude with weighting can distinguish patients at a group level. The increase of tremor amplitudes in PsyT may arise from increased coactivation to maintain oscillation.^3^ However, we found a large variability in TP changes in our OrgT cohort, so the upper limit of our normal range was set quite high (130%). Even so, 1 patient with NT and 1 with DT showed even larger increases of TP, which may simply reflect fluctuations in tremor amplitude in DT (unrelated to loading) and may be a consequence of muscle weakness in NT.

A small proportion of PsyT patients (5 of 13) entrained to one of the three requested tapping frequencies, which was not seen in any OrgT patients. A significant frequency shift (but not true entrainment) was considerably more common (10 of 13 PT patients). These data indicate that true entrainment is perhaps quite uncommon, in contrast to significant absolute changes in tremor frequency and marked intraindividual variability with tapping.^7^ Recording of more than one tapping frequency is useful, because it increases the chance to detect entrainment or a significant frequency shift. We confirm that recording the tapping performance is mandatory,^7^ because 6 of 13 PsyT patients had an incorrect task performance during at least one of the tapping frequencies. A pathological frequency shift, which is most likely to be the result of distraction, is clearly less likely to occur in patients who do not pay enough attention to correctly perform the tapping task.

An early study using time-frequency analysis found a frequency dissociation in bilateral tremors in 9 of 12 PD and 4 of 7 ET patients, but in neither of 2 patients with PsyT.^8^ Because these investigators also found that tremor either stopped or entrained with contralateral tapping in 5 of 5 PsyT patients, they concluded that PsyT affecting multiple limbs has only one oscillator, and that the coexistence of muscle groups phasically contracting at different frequencies is evidence against a psychogenic tremor aetiology.^8^ However, in line with our findings, a more recent study investigating 15 PsyT patients with bilateral tremor using coherence analysis found that only 7 patients had significant coherence between the two hands, whereas 8 patients had independent contralateral tremor rhythms.^11^ Two possible mechanisms that have been suggested to underlie noncoherent PsyT are a clonus mechanism and tremor that is so “overtrained” that it runs automatically and is not perturbed by another voluntary movement.^6^, ^11^

The myographic equivalent of the clinical “coactivation sign” is a short (i.e., ∼300 ms) tonic coactivation phase before the onset of tremor bursts.^3^ We were not able to capture the onset of tremor in all patients, because tremor was continuous in some. We confirm that the presence of a tonic coactivation phase before tremor onset is characteristic of PsyT and corresponds, in most cases, with the presence of clinical coactivation, but on blinded assessment, test sensitivity was only 46%.

There is a problem inherent in patient selection for studies such as this where current diagnostic criteria are based, in part, on items of which one is hoping to test specificity and sensitivity. This runs the risk of a circular argument, that patients are selected with a diagnosis of PsyT because (for example) tremor is distractible and entrainable, and then the specificity of distractibility and entrainability as a diagnostic feature of PsyT is assessed. This is an important issue that has not always been acknowledged in previous studies. We tried to overcome this problem by also including patients in whom the diagnosis of PsyT was based on features not relevant to the electrophysiological tests under investigation. Another potential problem is the fact that there is no 100% certainty that the clinical diagnosis of PsyT is correct in all cases; hence, a very small chance remains that clinical misdiagnosis may explain part of the variability in the electrophysiological findings.

The clinical presentation of PsyT varies widely, and the underlying mechanisms leading to tremor generation may be heterogeneous.^11^ It is, therefore, not surprising that our data show that one single laboratory test alone seems insufficient to correctly separate PsyT from OrgT. We have shown that in our cohort, patients with PsyT may be identified by a varying subset of tests (Table 4). We have also demonstrated that well-characterized patients with different forms of OrgT can have abnormalities on certain electrophysiological tests said to distinguish PsyT from OrgT. However, by using a combination of tests, we can fully discriminate this cohort of PsyT and OrgT patients. We do not claim that the results of this study have predictive value as yet, because all patients were clinically diagnosed before assessment. Also, current data most likely overestimate the discriminative potential of the sum score as it was applied in the same sample used for test selection. However, the current study does provide evidence to support a select group of simple electrophysiological tremor studies whose sensitivity and specificity can now be tested prospectively in a larger multicenter trial including an unselected group of patients with tremor. The equipment required for these tests, and testing time necessary, is limited. If the results here are confirmed, then this simple battery of tests may be suitable to provide a laboratory-supported level of certainty in the diagnosis of PsyT.
