# Supplementary material for: Moving Toward “Laboratory-Supported” Criteria for Psychogenic Tremor
Source: Mov Disord. 2011 Sep 28;26(14):2509–15. doi: 10.1002/mds.23922 (PMC3664413; doi:10.1002/mds.23922)
Supplement: Supplementary file 1 [file mds0026-2509-SD1.doc]

Supplementary Material

Table 5: Results of the test battery in 25 patients with organic tremor.

| OrgT | Diagnosis | Incorrect tapping | Response to tapping | Response to ballistic movements | Tonic coactivation | Coherence | Response to loading | **Total score (Max. 10)** |
| --- | --- | --- | --- | --- | --- | --- | --- | --- |
| 1 | PD | 1 | 0 | 1 | 0 | 0 | 0 | **2** |
| 2 | ET | 0 | 0 | 0 | 0 | 0 | 0 | **0** |
| 3 | DT | 0 | 0 | 0 | 0 | 0 | 0 | **0** |
| 4 | PD | 0 | 0 | 0 | 0 | 1 | 0 | **1** |
| 5 | PD | 0 | 0 | 1 | 0 | 0 | 0 | **1** |
| 6 | ET | 0 | 0 | 0 | 0 | 0 | 0 | **0** |
| 7 | DT | 0 | 0 | 0 | 0 | 0 | 0 | **0** |
| 8 | PD | 0 | 0 | 0 | 0 | NA | 0 | **0** |
| 9 | N T | 1 | 0 | 0 | 0 | 0 | 1 | **2** |
| 10 | DT | 0 | 1 | 1 | NA | 0 | 0 | **2** |
| 11 | DT | 0 | 0 | 0 | NA | 0 | 0 | **0** |
| 12 | ET | 0 | 0 | 0 | 0 | 0 | 0 | **0** |
| 13 | PD | 1 | 0 | 0 | 0 | 0 | 0 | **1** |
| 14 | ET | 0 | 0 | 0 | 0 | 0 | 0 | **0** |
| 15 | ET | 0 | 0 | 1 | 0 | 0 | 0 | **1** |
| 16 | PD | 0 | 0 | 0 | 0 | 0 | 0 | **0** |
| 17 | PD | 0 | 1 | 0 | 0 | 0 | 0 | **1** |
| 18 | DT | 0 | 0 | 0 | 0 | 0 | 0 | **0** |
| 19 | PD | 0 | 0 | 0 | 0 | NA | 0 | **0** |
| 20 | PD | 0 | 1 | 0 | 0 | 0 | 0 | **1** |
| 21 | DT | 0 | 0 | 0 | 0 | 0 | 0 | **0** |
| 22 | DT | 0 | 0 | 0 | 1 | 0 | 1 | **2** |
| 23 | DT | 0 | 0 | 0 | 0 | 0 | 0 | **0** |
| 24 | DT | 0 | 0 | 0 | 0 | 0 | 0 | **0** |
| 25 | NT | 1 | 0 | 0 | 0 | 0 | 0 | **1** |

Legend to Table 5: OrgT (Organic tremor); PD (Parkinson’s disease tremor); ET (Essential tremor); DT (Dystonic tremor); NT (Neuropathic tremor); NA (not applicable) indicates lack of a recording of tremor onset in continuous tremors (Tonic coactivation), presence of a unilateral tremor only (Coherence).
